# Development of Antifouling Coatings Based on Quaternary Ammonium Compounds through a Multilayer Approach

**DOI:** 10.3390/ijms24076594

**Published:** 2023-04-01

**Authors:** Denisa Druvari, Georgia C. Lainioti, Vlasoula Bekiari, Pavlos Avramidis, Joannis K. Kallitsis, Georgios Bokias

**Affiliations:** 1 Department of Chemistry, University of Patras, GR-26504 Patras, Greece; 2 Department of Food Science & Technology, University of Patras, GR-30100 Agrinio, Greece; 3 Department of Agriculture, University of Patras, GR-30200 Messolonghi, Greece; 4 Department of Geology, University of Patras, GR-26504 Patras, Greece

**Keywords:** antifouling coatings, aquaculture nets, cross-linked polymers, quaternary ammonium compounds, multilayer

## Abstract

The development of polymeric materials as antifouling coatings for aquaculture nets is elaborated in the present work. In this context, cross-linked polymeric systems based on quaternary ammonium compounds (immobilized or releasable) prepared under mild aqueous conditions were introduced as a more environmentally friendly methodology for coating nets on a large scale. To optimize the duration of action of the coatings, a multilayer coating method was applied by combining the antimicrobial organo-soluble copolymer poly(cetyltrimethylammonium 4-styrenesulfonate-co-glycidyl methacrylate) [P(SSAmC_16_-co-GMA20)] as the first layer with either the water-soluble copolymer poly(vinylbenzyl trimethylammonium chloride-co-acrylic acid) [P(VBCTMAM-co-AA20)] or the water-soluble polymers poly(acrylic acid) (PAA) and poly(hexamethylene guanidine), PHMG, as the second layer. The above-mentioned approach, followed by thermal cross-linking of the polymeric coatings, resulted in stable materials with controlled release of the biocidal species. The coated nets were studied in terms of their antifouling efficiency under accelerated biofouling conditions as well as under real conditions in an aquaculture field. Resistance to biofouling after three water-nutrient replenishments was observed under laboratory accelerated biofouling conditions. In addition, at the end of the field test (day 23) the uncoated nets were completely covered by marine contaminants, while the coated nets remained intact over most of their extent.

## 1. Introduction

Marine biofouling is an undesirable process in which micro- and macro-organisms attach to any object submerged in the sea and its adverse effects are reflected in the economy, ecology and shipping industries [1]. In the aquaculture industry, biofouling is one of the main obstacles to efficient and sustainable production [2]. The growth of aquatic species, affects shellfish, fish and seaweed farming worldwide, with the direct economic costs of managing biofouling in the aquaculture industry estimated at 5–10% of production costs [3]. The growth of algae, barnacles and bivalves can cover up to 100% of the mesh openings in cage nets and lead to net deformation and volume reduction. In addition, health of farmed fish is directly affected by biofouling, as they are exposed to pathogens associated with the fouling organisms, while water exchange is also impeded thereby reducing oxygen levels [4].

Every natural and artificial substrate in the marine environment is rapidly colonized by various species of micro- and macro-organisms. In general, researchers have defined four phases of colonization of marine biological contaminants: adsorption of dissolved organic molecules (molecular biofouling), colonization by prokaryotes, colonization by unicellular eukaryotes (diatoms, flagellates, amoebae and ciliates) and recruitment of invertebrate larvae and algal spores [5,6]. These phases may occur sequentially, overlap or take place simultaneously [7]. Nevertheless, some correlation may be shown, since the early phases, which are the adsorption of molecules and the colonization by prokaryotes, are crucial for the later phases, which are the settlement of larvae and spores.

Biofouling control in aquaculture comprises: the prevention of the primary establishment of contaminants by repelling or killing them, the inhibition of the development of established organisms by lowering their adhesion ability or by removing them when small and immature and the removal or elimination of established growth of biological foulants. The main strategies used to date for tackling marine biofouling are divided in two broad categories: antifouling coatings (AF) and fouling release coatings (FR) [8]. The performance of FR coatings is based mostly on hydrophobic surfaces which prevent the adhesion of biofoulants to the surface due to their low surface energy [9]. The most used materials for such surfaces are based on polydimethylsiloxane (PDMS) [10,11] and fluorinated materials [12] which are generally proven to be highly effective against biofouling. On the other hand, AF coatings are based on the impregnation and subsequent release of active biocides such as copper and organotin compounds which despite their high efficiency, they have raised concern worldwide due to their high toxicity levels [13,14]. For example, Kartal et al. [15] encapsulated and impregnated an eco-friendly antifouling chemical, econea active agent, to the ultra-high-molecular-weight polyethylene (UHMWPE) fishing nets using polyurethane and acrylic as binders. The antifouling activities of encapsulated econea showed high durability in the fish farm environment, open pores of the fishing nets and no viable proliferation when polyurethane was used as binder. In another work [16], nano-ZnO, Cu_2_O, zinc borate, and Econea multilayer films were applied to UHMWPE fishing nets through the Layer by Layer technique to enhance their antifouling properties. The nets covered with the Econea/PDDA_16_ (diallyldimethylammonium chloride) nanoparticles prevented biofouling and mesh occlusion on the nets compared to the other coatings. Frydenberg et al. [17] encapsulated copper pyrithione (CuPT) crystals by silica aerogel to obtain loadings of 50−80 % wt. CuPT. The results showed that the spatial confinement of CuPT crystals and the strong attachment to the coating enable the air gel to maintain a controlled release of dissolved CuPT for a prolonged time period. Cao et al. [18] fabricated an antifouling surface via coating Urgency BMox2 (TB) onto dopamine-modified 304 stainless steel (304 SS) and found that the adhesion rates of *Vibrio natriegens* and *Phaeodactylum tricornutum* on the antifouling surface were reduced by 99.85% and 67.93%, respectively, from those of untreated samples.

To improve the fouling resistance of AF coatings, other research approaches are focusing recently on the exploration of amphiphilic systems by introducing hydrophilic polymers, zwitterionic polymers [19,20,21] or quaternary ammonium compounds (QACs) [22,23,24,25,26]. Within this frame, in our laboratory we are focusing on the development of antifouling coatings based on cross-linked polymeric networks which bear QACs [27,28,29]. More specifically, synthesized antimicrobial copolymers bearing immobilized 4-vinylbenzyldimethylhexadecylammonium chloride (VBCHAM) biocidal groups and 4-styrenesulfonate cetyltrimethylammonium (SSAmC_16_) units with releasable cetyltrimethylammonium (AmC_16_) biocidal groups, were mixed and cross-linked in organic solvents, providing stable coatings with long-lasting antifouling efficiency. Furthermore, towards to a greener approach, some first attempts to prepare water-based antifouling coatings were recently presented [30], where immobilized quaternary ammonium groups with a short aliphatic chain acted effectively against biofouling. However, these coatings did not show long duration of action when tested under accelerated laboratory conditions.

The aim of the present work was to develop a methodology for the preparation of antifouling coatings with increased long-lasting activity, based on the use of water mostly as solvent, which could lead to a facile fabrication approach for marine industries, while maintaining green characteristics. Hence, having in mind the L-b-L (layer-by-layer) concept, we herein present a multilayer coating method of fishing nets, taking advantage of our previous knowledge on thermal cross-linking reaction of complementary carboxyl and epoxy groups of acrylic acid (AA)-containing copolymers and glycidyl methacrylate (GMA)-containing copolymers, respectively. For the first layer, the antimicrobial copolymer poly(cetyltrimethylammonium 4-styrenesulfonate-co-glycidyl methacrylate) [P(SSAmC_16_-co-GMA20)] was used, dissolved in dimethyl sulfoxide (DMSO). DMSO is an organic solvent widely used in several applications due to its less toxic nature, compared to other organic solvents [31]. The water-soluble copolymer poly(vinylbenzyl trimethylammonium chloride-co-acrylic acid) [P(VBCTMAM-co-AA20)], which was synthesized in a previous work [30], was used as the second layer, and it was stabilized through the thermal cross-linking reaction. Alternatively, an aqueous solution of poly(acrylic acid) (PAA) or a mixture of PAA with the biocidal poly(hexamethylene guanidine hydrochloride) (PHMG) was used as the second layer of the coated nets. PHMG was chosen for this study since it is well known for its wide range of action against bacteria, fungi and viruses, combined with antifouling activity against macro-pollutants in the aquatic environment [32,33,34]. The application of this protocol once or twice results in two-layer or four-layer coatings, respectively. The prepared coatings were studied in terms of their release ratio in aqueous conditions as well as their antifouling effectiveness in accelerated and real conditions, using a scale up process for coating aquaculture nets.

## 2. Results and Discussion

### 2.1. Characterization of Polymers

Poly(hexamethylene guanidine hydrochloride), PHMG, was prepared via condensation polymerization of hexamethylenediamine and guanidine hydrochloride.

**Figure 1:**
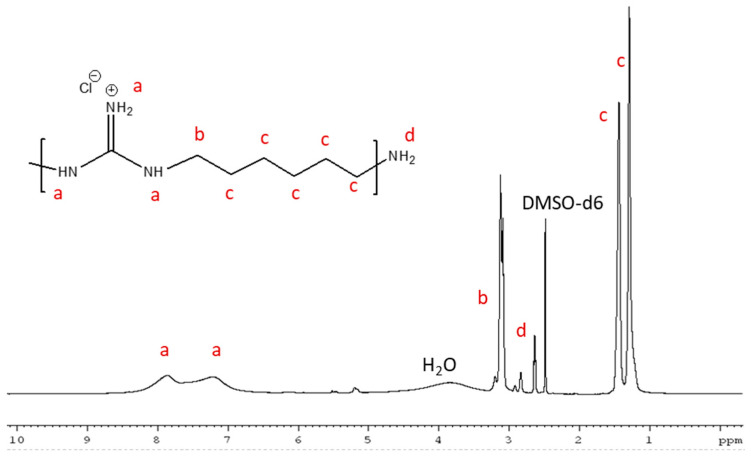
^1^H-NMR spectrum of PHMG oligomer in DMSO-d6 deuterated solvent.

The structural characterization of PHMG oligomer, was conducted through ^1^H-NMR spectroscopy in deuterated DMSO-d6. The respective spectrum and the attribution of the chemical shifts of the protons derived from PHMG are shown in Figure 1. In particular, the protons of the -CH_2_- methyl groups (c) appear at the 1.2–1.5 ppm region, the two protons of the -CH_2_-NH- group (b) at 3.1 ppm, the guanidine -NH_2_- amino groups (a) at 7.0–8.1 ppm and the two protons of the terminal amino group (d) appear at 2.7 ppm.

**Figure 2:**
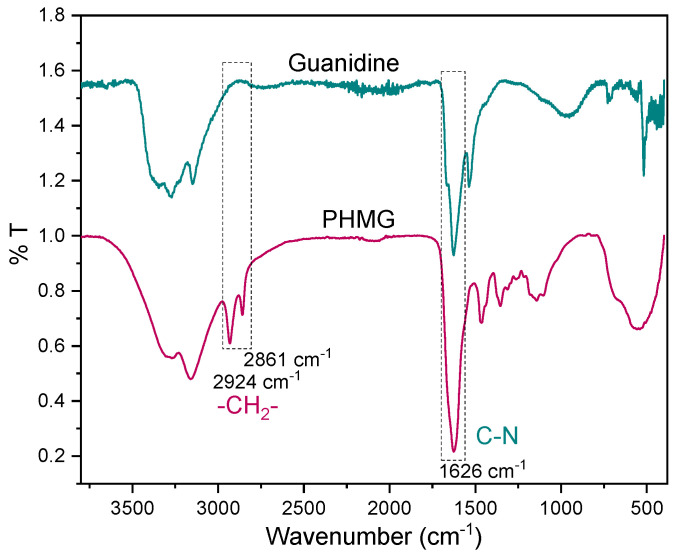
ATR-FTIR spectra of guanidine and the PHMG oligomer obtained after polycondensation.

The success of the polycondensation of guanidine salt with hexamethylene diamine was verified by ATR-FTIR spectroscopy, as shown in Figure 2. In the PHMG spectrum, the presence of the guanidine group is evidenced through the main peak at 1626 cm^−1^, attributed to the C=N double bond vibrations, as well as the broad peaks with maxima at about 3150 and 3280 cm^−1^, corresponding to the N-H stretching vibrations of guanidine bonds, as well as the methylene groups. The presence of a hexamethylene unit is attested by the two peaks at 2924 and 2861 cm^−1^, attributed to the vibrations of the methylene -CH_2_- group.

The molecular weight of the oligomer was found to be *Μ***_η_**: 646, using the Mark–Houwink–Sakurada equation: [*η*] = *K* × *M^a^*, where the values of *K* and *α* are known from the literature [35]. Therefore, given that the molecular weight of the structural unit of PHMG is 158, the oligomer appears to be composed of four structural units of hexamethylene guanidine hydrochloride. The results are shown in Table 1.

### 2.2. Preparation of Coated Nets

The multilayer coating concept was applied for the present polymeric systems because it offers several advantages. On the one hand, the combination of the desired organo-soluble copolymer with the water-soluble polymers in two (or four) consecutive coatings is easily achieved, thus avoiding the case of poor miscibility of the polymer solutions. On the other hand, in this way the biocide AmC_16_ is expected to be trapped more effectively in the inner polymeric layer(s), resulting in a slower release rate in the marine environment, thus, providing possibly a long-term antifouling performance.

**Figure 3:**
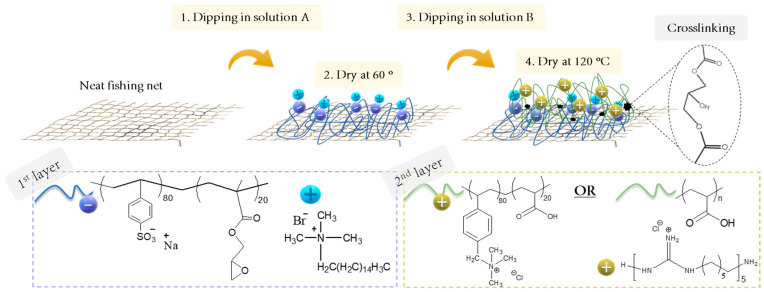
Multilayer coating of aquaculture nets with antimicrobial cationic polymeric materials.

In this line, the net was first immersed in a solution of P(SSAmC_16_-co-GMA20) providing the first coating. Regarding the second layer, two alternative options of water-soluble cationic polymeric materials were used. The first one was the P(VBCTMAM-co-AA20) copolymer which combines immobilized trimethylammonium groups with possible antimicrobial activity [36], with carboxyl groups of acrylic acid which can be used for further cross-linking reaction with the first polymeric layer, P(SSAmC_16_-co-GMA20), after heat treatment at 120 °C, as shown in Figure 3. The second option consisted of the water-soluble homopolymer PAA, which was used either alone, as a hydrophilic outer coating that would stabilize the whole system through cross-linking with GMA and potentially act as a fouling-resistant surface, or synergistically with the cationic oligomer PHMG, which provides strong antimicrobial activity. The mol/mol ratio of PAA/PHMG polymers in the mixture was 1/2. Table 2 shows the three different cases of two-layer coated nets, the *w*/*w* ratios of the two layers and the total percentage of polymeric loading on each net, as determined gravimetrically. SEM characterization reveals that the nets were quite uniformly coated (Figure S1), while the presence of atoms characteristic of the two layers was verified through EDS (Figure S2).

**Figure 4:**
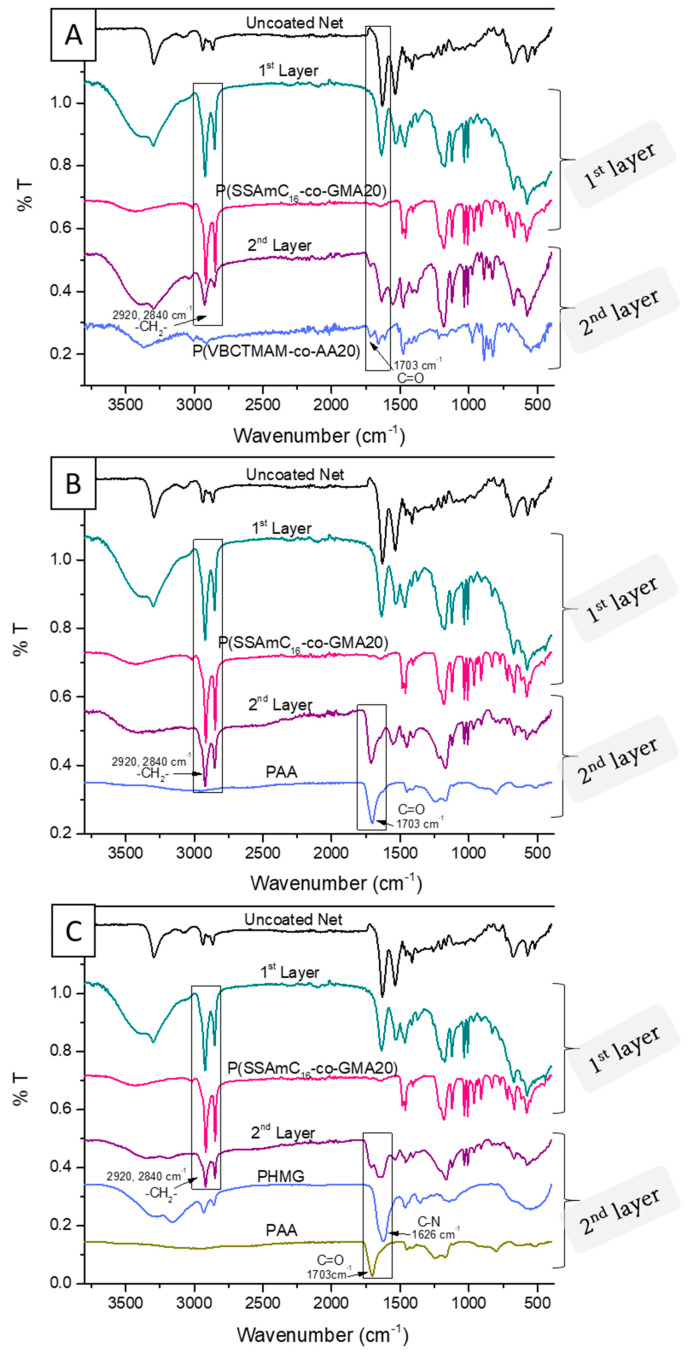
ATR-FTIR spectra of coated nets with CN1 (**A**), CN2 (**B**) and CN3 (**C**). The spectra of the individual polymers as well as the uncoated net are also shown for comparison.

Subsequently, characterization of the coated nets was carried out by ATR-FTIR spectroscopy, through which the successful modification of the nets was certified. More specifically, Figure 4A shows the spectra of the nets modified with the CN1 system: P(SSAmC_16_-co-GMA20)/P(VBCTMAM-co-AA20) 55/45 % wt. The graph also shows the spectra of P(SSAmC_16_-co-GMA20), P(VBCTMAM-co-AA20) copolymers and the uncoated net for comparison. As can be seen, the characteristic peaks of the individual copolymers are also observed in the spectra of the net after each coating. Indicatively, in the first layer the peaks of the methyl groups of SSAmC_16_ are observed at 2920 and 2840 cm^−1^, while in the second layer a peak at 1703 cm^−1^ owed to the carbonyl group of acrylic acid is observed. Similarly, in the spectra of CN2: (P(SSAmC_16_-co-GMA20)/PAA20 70/30 % wt. (Figure 4B), the same peaks at 2920 and 2840 cm^−1^ of the first layer are shown, while in the second layer a peak at 1703 cm^−1^ attributed to the carbonyl group of PAA is also shown. In the spectra of the net CN3: P(SSAmC_16_-co-GMA20)/PAA/PHMG 70/30 % wt. (Figure 4C), the same characteristic peaks of the polymers P(SSAmC_16_-co-GMA20) and PAA are observed (2920, 2840 and 1703 cm^−1^). Furthermore, a new peak at 1626 cm^−1^ is shown, which is attributed to the C=N vibrations of PHMG on the outer layer.

### 2.3. Release Study of Polymer Coatings

For each cycle the soluble fraction of the polymeric material % wt. and the solvent uptake % wt. were determined. The results are shown in Table 3.

From the results of Table 3 we may draw the conclusion that all systems appear more stable in salt solution than in water, as they show very low soluble fraction values (from 2 to 9 % wt.), with CN1 net (P(SSAmC_16_-co-GMA20)/P(VBCTMAM-co-AA20)) showing significantly higher mass loss in pure water (32–35 % wt.) than the other systems (5–15 % wt.). In terms of solvent uptake, CN1 net also showed higher values in pure water in comparison to CN2 and CN3. This observation, associated with the high mass loss, probably indicates that the system is loosely cross-linked (high solvent uptake) and a high percentage of uncross-linked P(VBCTMAM-co-AA20) polymer chains are, in fact, present (high mass loss) in the outer layer (55/45 % wt.). A general observation is that the solvent uptake in salt solution is similar or even higher than that observed in pure water. In particular, the CN2 (P(SSAmC_16_-co-GMA20)/PAA) net also shows a high solvent uptake in the salt solution (190 % wt.) while its mass loss was very low (4 % wt.). This observation is encouraging for the application of the coating in real seawater conditions, as the increased solvent uptake of the PAA layer suggests that it could possibly act as a highly hydrated antifouling surface.

**Figure 5:**
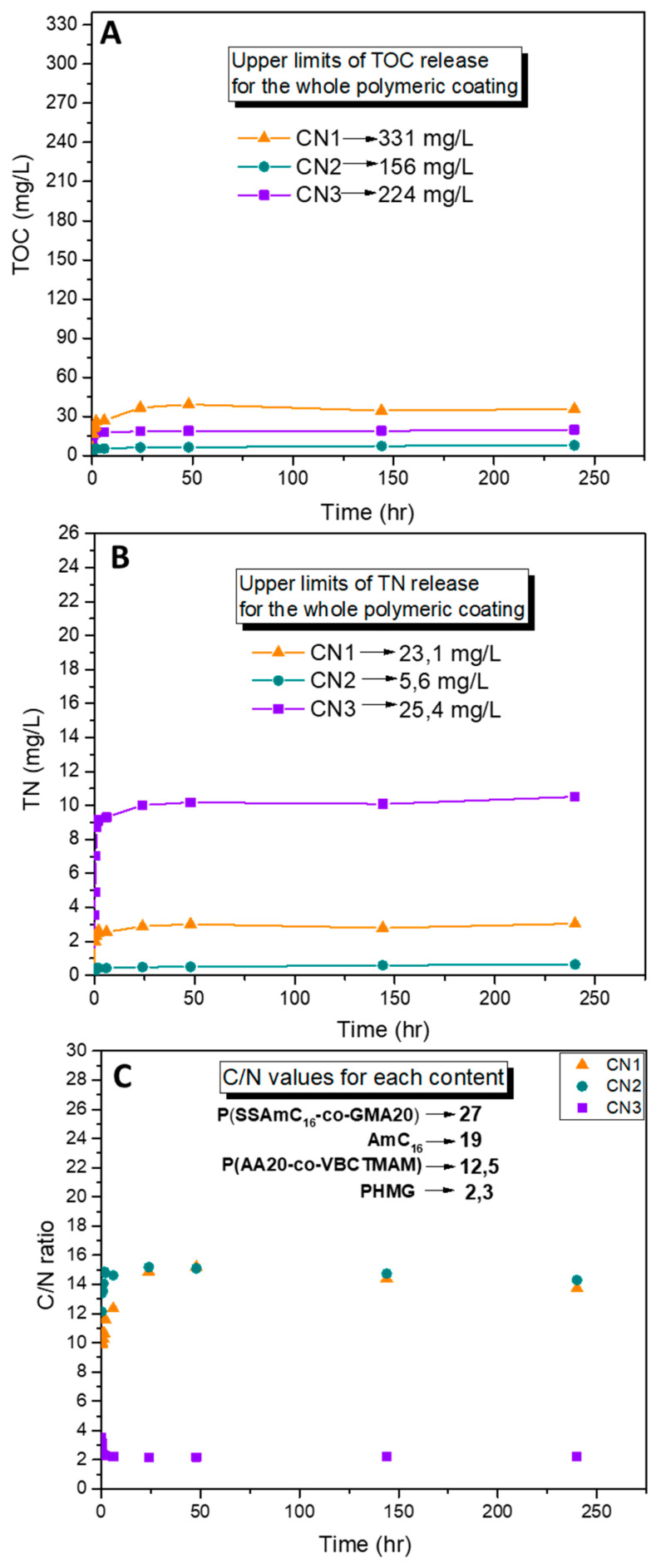
Measurements of (**A**) Total Organic Carbon (TOC), (**B**) Total Nitrogen (TN) and (**C**) atomic C/N r ratio as a function of time after immersion of coated nets CN1, CN2 and CN3 in 0.6 M NaCl solution for 10 days (1st cycle).

In an attempt to better understand the behavior of coated nets, the release of structural groups from the coatings was evaluated using Total Organic Carbon (TOC) and Total Nitrogen (TN) measurements. For this study, the nets in Table 3 were immersed only in aqueous 0.6 M NaCl solution, as the final application will be in seawater of a similar salinity. As shown in Figure 5A–C, regarding the first release cycle lasting 10 days, the TOC and TN values of the three coatings CN1, CN2 and CN3 remained at low levels until the end of the experiment, as compared with the TOC, TN values calculated assuming that the entire polymer coating could be dissolved. The low TOC, TN values observed in the first ten days are qualitatively in agreement with the relatively low mass loss observed in saline solution.

Figure 5C shows the atomic C/N ratio values calculated from the TOC, TN measurements for each coating, as well as the theoretical values obtained for each releasable group or polymer that could potentially be dissolved. According to the overall results, we can draw a more confident conclusion about the type of released material. For the coated net CN1, a low release of the outer coating P(VBCTMAM-co-AA20) is observed as indicated by the atomic C/N ratio (~11–14). In the case of CN2 net, a very low release is also observed. Though the atomic C/N ratio (~14–16) is somewhat underestimated, due possibly to the low TN values, the release of AmC_16_ groups (C/N ratio = 19) is most probable in this case, since much higher values (>27) should be observed if the whole inner layer ((P(SSAmC_16_-co-GMA20)) or the whole coating was releasable. In the case of the CN3 net, on the other hand, a higher release is observed in this first cycle, as the TOC and TN values are higher, while, according to the C/N value (~2), PHMG is immediately released. These values are due to the excess of PHMG contained in the PAA/PHMG outer layer mixture, which apparently is not retained through some special interaction with the other polymers and is quickly released.

**Figure 6:**
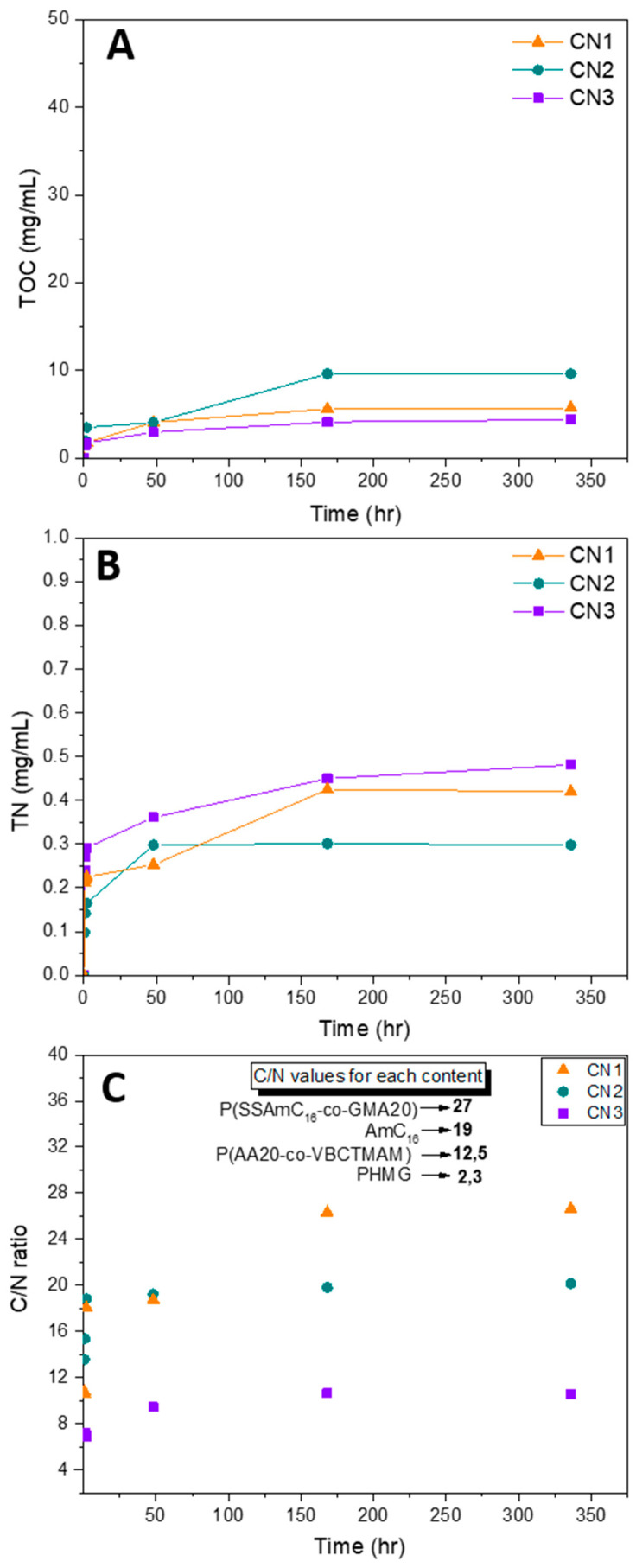
Measurements of (**A**) Total Organic Carbon (TOC), (**B**) Total Nitrogen (TN) and (**C**) atomic C/N ratio as a function of time after immersion of coated nets CN1, CN2 and CN3 in aqueous 0.6 M NaCl solution for 15 days (2nd cycle).

After 10 days the nets were transferred to a new aqueous 0.6 M NaCl solution, where they remained for a second 15-day cycle and the new TOC, TN and C/N measurements (after 25 days) are shown in Figure 6A–C. During the second cycle of the release study, even lower TOC and TN values were observed in all coatings, demonstrating the continued slow release of biocidal materials, leading to controlled release systems. Though determination uncertainties are important as a consequence of the marginal TN values measured, it is clear that only the P(VBCTMAM-co-AA20) copolymer is released from the CN1 in the first 50 h, and then the biocidal bottom layer copolymer, P(SSAmC_16_-co-GMA20) (C/N~27), begins to be released as well. Moreover, the CN2 net shows very low release of the biocide AmC_16_ (C/N~19), while the CN3 net this time shows low release which is probably due to both biocides, PHMG and AmC_16_.

In conclusion, the release study through gravimetry and TOC/TN measurements revealed a promising behavior of the coated nets under aqueous conditions, especially under simulated seawater conditions (NaCl 0.6 M). These results are a guide for the expected antifouling effect of the coatings when applied to fish farming nets and immersed in seawater.

### 2.4. Action of New Polymeric Coatings against Biofouling

#### 2.4.1. Testing under Accelerated Biofouling Conditions

**Figure 7:**
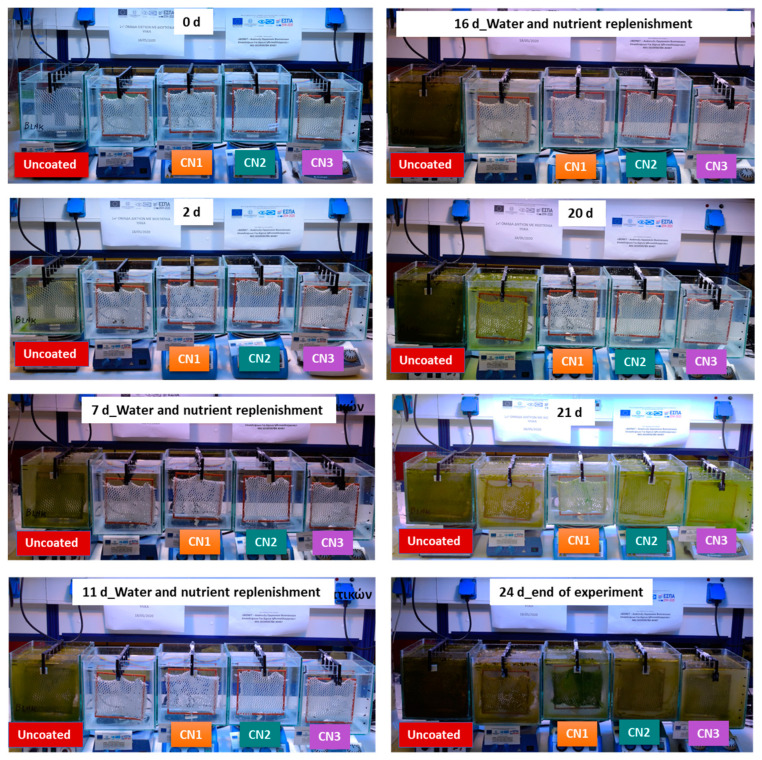
Study of the polymeric coatings against biofouling after immersion in seawater for 24 days under accelerated conditions. Water and nutrient replenishment were carried out at intermediate time intervals.

The next step was to test the coated nets with the above-mentioned stable polymeric systems for their anti-fouling performance as a function of time. Initially, tests were carried out on a laboratory scale and under accelerated biofouling conditions. In this line, the nets presented in Table 2 were immersed in aquariums with seawater to which nutrients were added to enhance the growth of bio-foulants (algae, etc.). Knowing that the growth cycle of biofouling takes six to seven days to be completed, the seawater-nutrient solution was replenished three times over a 24-day period, when the experiment was terminated. As shown in Figure 7, the uncoated net presented biofouling from the very first days. Specifically, on day 11, the net was full of fouling, while the surrounding water exhibited a dark green color and high turbidity. In contrast, nets CN1 (P(SSAmC_16_-co-GMA20)/P(VBCTMAM-co-AA20)), CN2 (P(SSAmC_16_-co-GMA20)/PAA) and CN3 (P(SSAmC_16_-co-GMA20)/PAA/PHMG) showed successful resistance to biofouling up to day 20 despite the three water-nutrient replenishments. At the end of the experiment (day 24), a dark green alga was observed on the surface of the CN1 net coated with P(SSAmC_16_-co-GMA20)/P(VBCTMAM-co-AA20), suggesting that the fouling organisms may still be active on the surface of the net, probably due to the high percentage of immobilized biocide groups on the coating that do not allow detachment of the inactive organisms. In this way, the accumulation of dead organisms on the surface acts as a secondary pollution-source which maintains the biofilm formation. On the other hand, in the cases of CN2 (P(SSAmC_16_-co-GMA20)/PAA) and CN3 (P(SSAmC_16_-co-GMA20)/PAA/PHMG) nets, the brownish color of alga indicates that the action of the hydrophilic PAA coating as a resistant-surface for the organisms, in synergy with the gradual release of the biocidal AmC_16_ into the water, exhibits a more effective activity against biofouling. The same phenomenon is also observed for the PAA/PHMG coating, but without showing any improvement in the overall activity.

**Figure 8:**
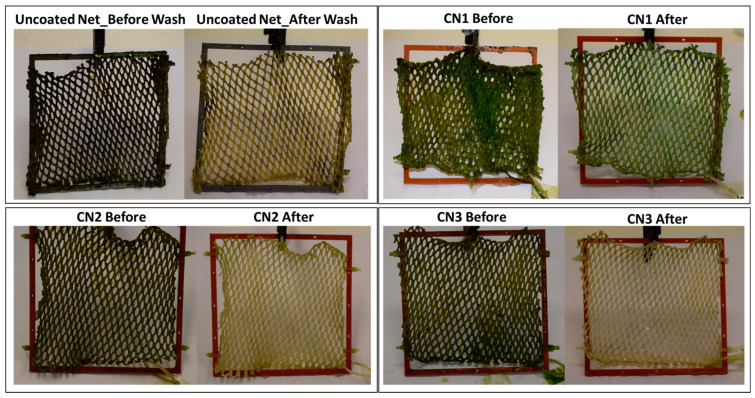
Photographs of the uncoated and coated nets before and after pressure washing with water, on day 24 of the experiment.

To avoid the harmful effects of biofouling development, the nets used in aquaculture are periodically cleaned with high-pressure washers. Following this line, the nets used in this study after their removal from the aquariums were washed with high-pressure water in the laboratory. Photographs of the nets before and after washing are shown in Figure 8. As can be seen, the cleaning efficiency of the uncoated net and the coated CN1 (P(SSAmC_16_-co-GMA20)/P(VBCTMAM-co-AA20)) net was not sufficient, in contrast to the other two coated nets CN2 (P(SSAmC_16_-co-GMA20)/PAA) and CN3 (P(SSAmC_16_-co-GMA20)/PAA/PHMG). More specifically, contaminants were easily removed from these nets, which showed significant difference before and after washing. From these results we can conclude that the coated nets CN2 and CN3 showed very weak fouling adhesion making them good candidates for possible use as a coating in aquaculture nets that will improve their life expectancy in marine applications.

A crucial question for the potential application is whether the nets can be repeatedly coated after use. To check this point, the net CN1, after application under accelerated laboratory conditions and washing with high pressure water, was re-coated using the same procedure. From the weight change a significant loading was verified, while the presence of the materials of both layers was revealed through ATR-FTIR characterization (Figure S3).

#### 2.4.2. Testing under Real Field Conditions

The final aim was to test the effectiveness of the new materials under real conditions, in the field. Therefore, the aforementioned polymeric coatings were applied to larger nets (30 × 20 cm) applying the methodology described earlier. To achieve a longer duration of action, replicate nets were coated for each system were coated twice (four-layer coatings) to attain a higher percentage of total loading (Table 4). Thus, for each system studied, two nets with different percentages of coating (two-layer and four-layer coating) and one uncoated net were used, which were fitted in a special circular arrangement placed in an aquaculture cage and immersed in the sea for 23 days.

**Figure 9:**
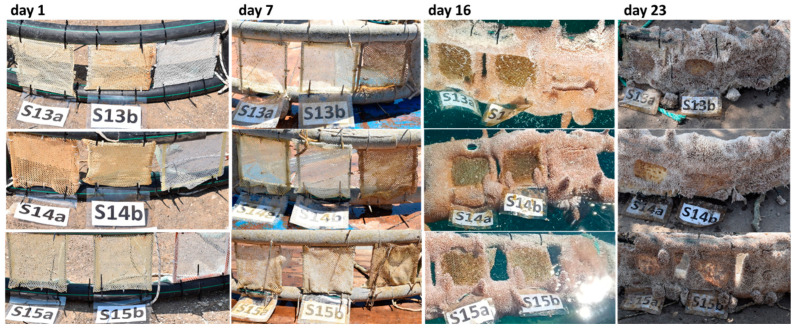
Coated nets with the polymeric systems P(SSAmC_16_-co-GMA20)/PAA (S13), P(SSAmC_16_-co-GMA20)/PAA/PHMG (S14) and P(SSAmC_16_-co-GMA20)/P(VBCTMAM-co-AA20) (S15), after immersion in the sea for various time periods. Every triplet of nets consists of an uncoated net (to the right) for comparison.

As shown in Figure 9, the uncoated nets started to exhibit fouling from day 7 of immersion, while the coated nets appeared to be more resistant. However, the difference between coated and uncoated nets was clear at the end of the field test (day 23), where the uncoated nets were completely covered by fouling organisms, while the coated nets still had intact, clean areas. Regarding the activity of the studied coatings, as can be observed, the coated nets showed equally high performance in all cases until the end of the test. More specifically, the nets were free of contaminants until day 16, while on day 23 they were partially coated, with nets S13b and S14a showing a slightly higher clean surface area. This observation is consistent with the results of the accelerated experiment conducted in the laboratory (Figure 7). Furthermore, it is clear that the increased loading percentage enhanced the efficiency of the respective polymer system.

In conclusion, the combination of organo-soluble P(SSAmC_16_-co-GMAx) copolymers with the water-soluble P(VBCTMAM-co-AAx) copolymers or water-soluble PAA/PHMG polymers by using the L-b-L coating concept, resulted in coatings with high antifouling activity, which may be owed to three reasons. First, the cationic releasable compounds (SSAmC_16_, PHMG) in the polymeric material impart a killing activity via biocide-release. Second, the cationic immobilized compounds (VBCTMAM) contribute through a contact-kill action. Third, the outer layer consisting of PAA acts as a fouling-resistant surface for fouling organisms due to its increased hydrophilicity and low surface charge [37]. According to the above results, the present study broadens the research on multiple polyelectrolyte coatings towards the formation of highly active anti-fouling materials [38,39,40].

Finally, as it may be seen from the photographs of Figure 9, the growth of the pollutant microorganisms started from the cages and transferred to the nets due to the very close distance between them. Bearing in mind that the experiment was conducted during the summer season where there is high bioaccumulation, and that the growth of microorganisms is a dynamic phenomenon strongly influenced by environmental conditions, the movement of cage and nets as well as the presence of fish in the cage, we can conclude that the observed activity of the coated nets in this study is quite encouraging.

## 3. Materials and Methods

### 3.1. Materials

The homopolymer poly(acrylic acid) (PAA) Mw: 250,000, the monomers acrylic acid (AA), sodium 4-styrene sulfonate (SSNa), glycidyl methacrylate (GMA), 4-vinylbenzyl chloride (VBC), initiator azobisisobutyronitrile (AIBN), trimethylamine solution ~45% (*w*/*w*) in H_2_O (TMAM), the surfactant *hexadecyltrimethylammonium bromide (*CTAB**) hexamethylenediamine (98%), guanidine hydrochloride (99.5%), sodium chloride (NaCl) and hydrochloric acid (HCl), as well as deuterated dimethyl sulfoxide (DMSO-d6) were obtained from Sigma-Aldrich (Aldrich, Steinheim, Germany) and used as received. The solvent dimethyl sulfoxide (DMSO) was purchased from Fischer (Fisher Scientific UK Ltd., Loughborough, UK) and used as received. Ultrapure water was obtained through an SG device water purification unit. Nylon nets (HelNet S.A., Schimatari, Greece) with 8 mm square mesh size, 15 mm stretch mesh size and 430 g/m^2^ weight were used for coating.

### 3.2. Synthetic Procedures

#### 3.2.1. P(SSAmC_16_-co-GMA20) and P(VBCTMAM-co-AA20)

The copolymers P(SSAmC_16_-co-GMA20) and P(VBCTMAM-co-AA20) were synthesized as previously reported [27,30]. The copolymer P(SSAmC_16_-co-GMA20) was dissolved in DMSO and was always used as the first layer on the net, whereas the aqueous solution of P(VBCTMAM-co-AA20) copolymer was used as the second layer for the net.

#### 3.2.2. PHMG

**Scheme 1:**
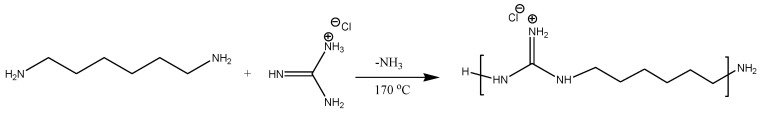
Synthesis of oligomeric PHMG via polycondensation reaction.

Hexamethylene guanidine hydrochloride oligomer (PHMG) was prepared according to the literature [41], via condensation polymerization. Equal amounts of hexamethylenediamine and guanidine hydrochloride were added to a three-necked spherical flask equipped with a thermometer and a freezer. The mixture was allowed to react first at 100 °C for 1 h and then at 170 °C for 4 h (Scheme 1). A special device containing an excess of HCl 1 M solution was connected to neutralize the ammonia gas produced. Ammonia gas started to evolve in the form of bubbles into the HCl 1 M solution after about one hour, indicating the start of the polycondensation reaction. The reaction was stopped after 5 h by cooling in an ice bath and the product was dissolved in water. A slightly yellow, viscous PHMG solution was obtained and the final product resulted in a solid via the freeze-drying process.

To determine the molecular weight of PHMG, the oligomer was dissolved in 0.3 M NaCl solution at various concentrations (2.0 to 8.0% *w*/*v*) and its intrinsic viscosity at 25 °C was determined using an Ostwald viscometer. The molecular weight of PHMG was calculated using the Mark–Houwink–Sakurada equation: [*η*] = *K* × *M^a^*, where the values of *K* and *α* are 0.46 × 10^−3^ (cm^3^ g^−1^) and 0.59, respectively, according to the literature [35].

### 3.3. Preparation of the Coated Aquaculture Nets

For the two-layer coating method, the P(SSAmC_16_-co-GMA20) solution in DMSO (10% *w*/*v*) was first prepared and used as the first layer of pre-weighed nets (15 × 15 cm) which were afterwards placed in an oven at 60 °C, for 24 h, for drying. Aqueous solutions of the polymers P(VBCTMAM-co-AA20) (4% *w*/*v*), PAA (15% *w*/*v*) and the PAA/PHMG mixture (10% *w*/*v*) were then prepared and used as the second coating of the nets. The mol/mol ratio of PAA/PHMG mixture was adjusted to 1:2, as excess of guanidinium groups was desired to enhance the biocidal activity of the coatings. Once the two polymers were mixed, a white solid was formed, probably due to the interactions (hydrogen bonds) between them, which was dissolved after vigorous stirring for a few minutes at room temperature (RT). After immersing the nets in the respective aqueous solutions for a short period of time, drying at 120 °C for 24 h followed, for stabilization through thermal and chemical cross-linking between the polymers. Coated nets were finally weighed in order to determine the loading of the coating which varied between 29–60 % wt.

This coating protocol was applied twice for the preparation of the four-layer coatings of the aquaculture nets.

### 3.4. Characterization Techniques

#### 3.4.1. Nuclear Magnetic Resonance Hydrogen Spectroscopy (^1^H-NMR)

The Bruker Avance DPX 400 spectrometer (Bruker BioSpin GmbH, Magnet Division, Karlsruhe, Germany) was used to study the materials in terms of their structure and composition. The ^1^H-NMR spectra were obtained at 400 MHz at 300 K. Deuterated chloroform (CDCl_3_) with internal tetramethylsilane (TMS) standard was used to dissolve the samples. For analysis of NMR data, the software Topspin-3.6.1 was used.

#### 3.4.2. Total Reflectance Fourier Transform Infrared Spectroscopy (ATR-FTIR)

ATR-FTIR spectra of coated and uncoated nets’ surfaces were obtained from Bruker Optics Alpha-P Diamond ATR Spectrometer of Bruker Optics GmbH (Ettlingen, Germany). ATR-FTIR spectroscopy was performed at room temperature in the range between 500–4000 cm^−1^.

#### 3.4.3. Scanning Electron Microscopy-Energy Dispersive Spectroscopy (SEM-EDS)

Scanning electron microscopy (SEM, Zeiss SUPRA 35VP instrument equipped with an EDS detector, Oberkochen, Germany) was performed to investigate the surface and the elemental analysis of uncoated and coated nets. The magnification level was 395–655 X.

### 3.5. Release Studies

#### 3.5.1. Soluble Fraction and Solvent Uptake Studies of Coated Nets

Small pieces of pre-weighed coated nets from each polymeric system were immersed in pure water as well as in 0.6 M NaCl aqueous solution and were left for a total period of 25 days divided into two cycles (10 and 15 days). The nets were removed at the end of each cycle, washed with pure water and dried at 80 °C for 24 h. The soluble fraction of the coated nets was then evaluated gravimetrically, as in each solvent, by the equation:

(1) $$\mathrm{Soluble}\;\mathrm{Fraction}\;(\%\;\mathrm{wt}.)=\;\frac{\left|W-W_{0}\right|}{W_{0}}\%$$

where *W*_0_ and *W* are the measured weights of the dry coatings before and after immersion in the solvents, respectively.

For the determination of solvent uptake, the nets were lightly wiped with filter paper after removed from the solvents and weighed immediately. Solvent uptake was calculated by the same equation mentioned above, where *W* is the measured weight of the wet coating after immersion in the respective solvents.

#### 3.5.2. Total Organic Carbon (TOC) and Total Nitrogen (TN) Measurements

Coated nets weighed approximately 0.2 g were immersed in pure water or in aqueous 0.6 M NaCl solution, depending on the experiment. At various time intervals, 1 mL quantities of the solutions were taken, diluted to 10 mL with respective solvent, and the TOC and TN content was measured. The volume of the quantity taken each time for the measurements was immediately replaced in order to maintain the total concentration. TOC and TN measurements were carried out according to the methodology described elsewhere [42] using a Schimadzu TOC analyzer (TOC-VCSH) coupled to a chemiluminescence detector (TNM-1 TN unit).

### 3.6. Antifouling Performance of Coated Nets

To test the activity of the coatings under accelerated biofouling conditions, the coated as well as the uncoated nets were placed in glass tanks filled with seawater, 3.5 mL of Walne nutrient solution and 3.5 mL of algae, to achieve the content: 1.0 mL of algae–nutrient mixture per liter of seawater. The tanks were placed under four multispectral lamps of 500 lux. The parameters inside the tanks were: Seawater temperature: 18.4–29.7 °C, pH: 8.17–9.88, dissolved O_2_: 5.63–16.88 ppm and salinity: 39.7–41.0 g/kg. Accelerated eutrophication conditions were visually examined in the uncoated net daily, ensuring that replicate conditions were obtained every fourth day from the start of the experiment.

Finally, a test of the antifouling effect of the coated nets was carried out in real field conditions. For this purpose, larger fish farming nets (30 cm × 20 cm) were used. For each studied system, two nets with different loading percentages and one uncoated net were used, which were fitted in a special circular arrangement placed into an aquaculture cage and submerged in the marine area of Selonda in the Saronic Bay of Greece, for 23 days.

## 4. Conclusions

In the present work a new greener methodology was developed for the preparation of anti-fouling coatings, based on aqueous polymeric systems which could be applied to aquaculture industry in a large scale. Active polymeric systems were prepared using the copolymer P(SSAmC_16_-co-GMA20), which had given encouraging results of antimicrobial activity with controlled release of biocidal species in a previous study [19]. Intending to prolong aquaculture coated nets’ activity, a multilayer-coating method was used to encapsulate the AmC_16_ biocidal groups in the inner layer of the net leading to their slow release into the marine environment. It is worth noting that for coating preparation, the solvent mixture H_2_O/DMSO was used, instead of other typical organic solvents (CHCl_3_, etc.) leading to a more environmentally friendly system, thus combining the use of organo-soluble P(SSAmC_16_-co-GMAx) copolymers (first layer) with water-soluble P(VBCTMAM-co-AAx) copolymers or water-soluble PAA and PAA/PHMG polymers (second layer). The release study of the coated nets through gravimetric and TOC/TN analysis showed generally good behavior of all coatings under aqueous conditions, especially under simulated seawater environment (NaCl 0.6 M). When tested under laboratory accelerated biofouling conditions, the coated nets showed resistance to biofouling successfully up to day 20 and after three water-nutrient replenishments. The most significant finding was in the final test of the coatings under real field test applications, where the coated nets remained clear up to day 16, compared to the uncoated nets. The difference was more striking at the end of the test (day 23), where the uncoated nets were completely covered by marine contaminants, while the coated nets remained intact over most of their extent. While this time is quite sufficient for some cases (for instance, fry production), the versatility of the present methodology offers several possibilities of optimization to attain longer effective periods, suitable for typical aquaculture applications. Though the proof-of-concept was demonstrated here using DMSO as the solvent of the first layer, in important aspect could be to avoid the use of any organic solvent and developing wholly water-based systems. Such a design could be feasible and offer possibilities to prolong the effectiveness of the coated nets through the control of interlayer depth and crosslinking density.

In conclusion, the combination of the organo-soluble P(SSAmC_16_-co-GMAx) copolymers with the water-soluble P(VBCTMAM-co-AAx) copolymers or water-soluble PAA/PHMG polymers in a two- (or four-) layer structure resulted in coatings with high antifouling activity. This may be owed to the presence of the cationic releasable groups (SSAmC_16_, PHMG) and immobilized cationic groups (VBCTMAM) in the polymeric material, imparting a biocide-release and contact-kill action, respectively, while the outer layer consisting of PAA acts as a fouling-resistant surface for contaminants. According to the above results, the new multi-functional and environmentally friendly polymeric coatings can be promising alternatives to address marine biofouling in aquaculture.

## Figures and Tables

**Table 1:** Results of the calculation of the *intrinsic* viscosity [*η*] and molecular weight *Μ***_η_** of the PHMG oligomer. The used Mark–Houwink–Sakurada parameters *K* and α have been found in literature [35].

Concentration (g/dL)	[*η*] (dL/g) in 0.3 N NaCl	*M*_η_
8.00	0.02095	646

**Table 2:** Coated nets with P(SSAmC_16_-co-GMA20)/P(VBCTMAM-co-AA20), P(SSAmC_16_-co-GMA20)/PAA or P(SSAmC_16_-co-GMA20)/PAA/PHMG layers.

Net Code	1st Layer (in DMSO)	2nd Layer (in H_2_O)	Layers’ Ratio % wt.	Coatings’ Loading % wt.
CN1	P(SSAmC_16_-co-GMA20)	P(VBCTMAM-co-AA20)	55/45	34
CN2	P(SSAmC_16_-co-GMA20)	PAA	70/30	30
CN3	P(SSAmC_16_-co-GMA20)	PAA/PHMG	70/30	35

**Table 3:** Soluble fraction % wt. and solvent uptake % wt. of the coated nets after immersion in water and aqueous 0.6 M NaCl solution for 25 days.

Net Code	Immersion Time	Soluble Fraction % wt.		Solvent Uptake % wt.	
		H_2_O	ΝaCl 0.6 M	H_2_O	ΝaCl 0.6 M
CN1	1st cycle (10 d)	32	6	167	159
	2nd cycle (25 d)	35	9	154	170
CN2	1st cycle (10 d)	5	4	30	190
	2nd cycle (25 d)	13	2	108	89
CN3	1st cycle (10 d)	14	6	80	130
	2nd cycle (25 d)	5	8	14	74

**Table 4:** Coating systems with different layers’ ratios and different percentages of total coating.

Net Code	1st (and 3rd) Layer (in DMSO)	2nd (and 4th) Layer (in H_2_O)	Number of Layers	Layers’ Ratio % wt.	Coatings’ Loading % wt.
S13a	P(SSAmC_16_-co-GMA20)	PAA	2	70/30	30
S13b	P(SSAmC_16_-co-GMA20)	PAA	4	70/30	50
S14a	P(SSAmC_16_-co-GMA20)	PAA/PHMG	4	70/30	60
S14b	P(SSAmC_16_-co-GMA20)	PAA/PHMG	2	70/30	29
S15a	P(SSAmC_16_-co-GMA20)	P(VBCTMAM-co-AA20)	2	55/45	30
S15b	P(SSAmC_16_-co-GMA20)	P(VBCTMAM-co-AA20)	4	55/45	43

## Data Availability

Data sharing not applicable.

## Supplementary Materials

The following are available online at https://www.mdpi.com/article/10.3390/ijms24076594/s1.
