# Supplementary material for: Development of Antifouling Coatings Based on Quaternary Ammonium Compounds through a Multilayer Approach
Source: Int J Mol Sci. 2023 Apr 1;24(7):6594. doi: 10.3390/ijms24076594 (PMC10094943; doi:10.3390/ijms24076594)
Supplement: Supplementary file 1 [file ijms-24-06594-s001.zip › ijms-2181853-supplementary.pdf]

Supplementary Material

Figure S1 shows the SEM surface morphology of the uncoated net, the net coated with the 1st layer, P(SSAmC$_{16}$-co-GMA20), and the nets CN1, CN3 coated with P(SSAmC$_{16}$-co-GMA20)/P(VBCTMAM-co-AA20) and P(SSAmC$_{16}$-co-GMA20)/PAA/PHMG, respectively. The figure shows the formation of a uniform coating over the nylon net.

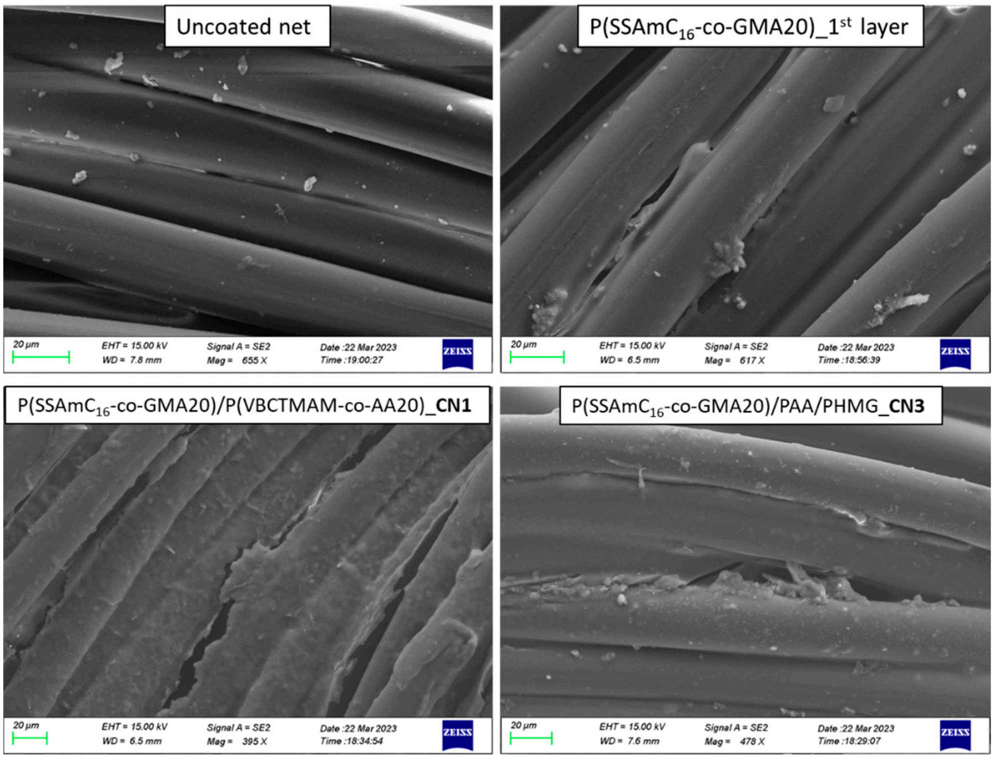

Figure S1. SEM images of the uncoated net, the net coated with the 1st layer, P(SSAmC$_{16}$-co-GMA20), and the nets CN1, CN3 coated with P(SSAmC$_{16}$-co-GMA20)/PAA and P(SSAmC$_{16}$-co-GMA20)/PAA/PHMG, respectively.

With the EDS analysis (Figure S2), it can be seen that S and Na appear in the net coated with the first layer, whereas on nets CN2 and CN3, Cl also appears which is the counter ion of P(VBCTMAM-co-AA20) and PHMG, respectively. The appearance of Pt is owed to a glue used for the stabilization of nets onto the sample holder.

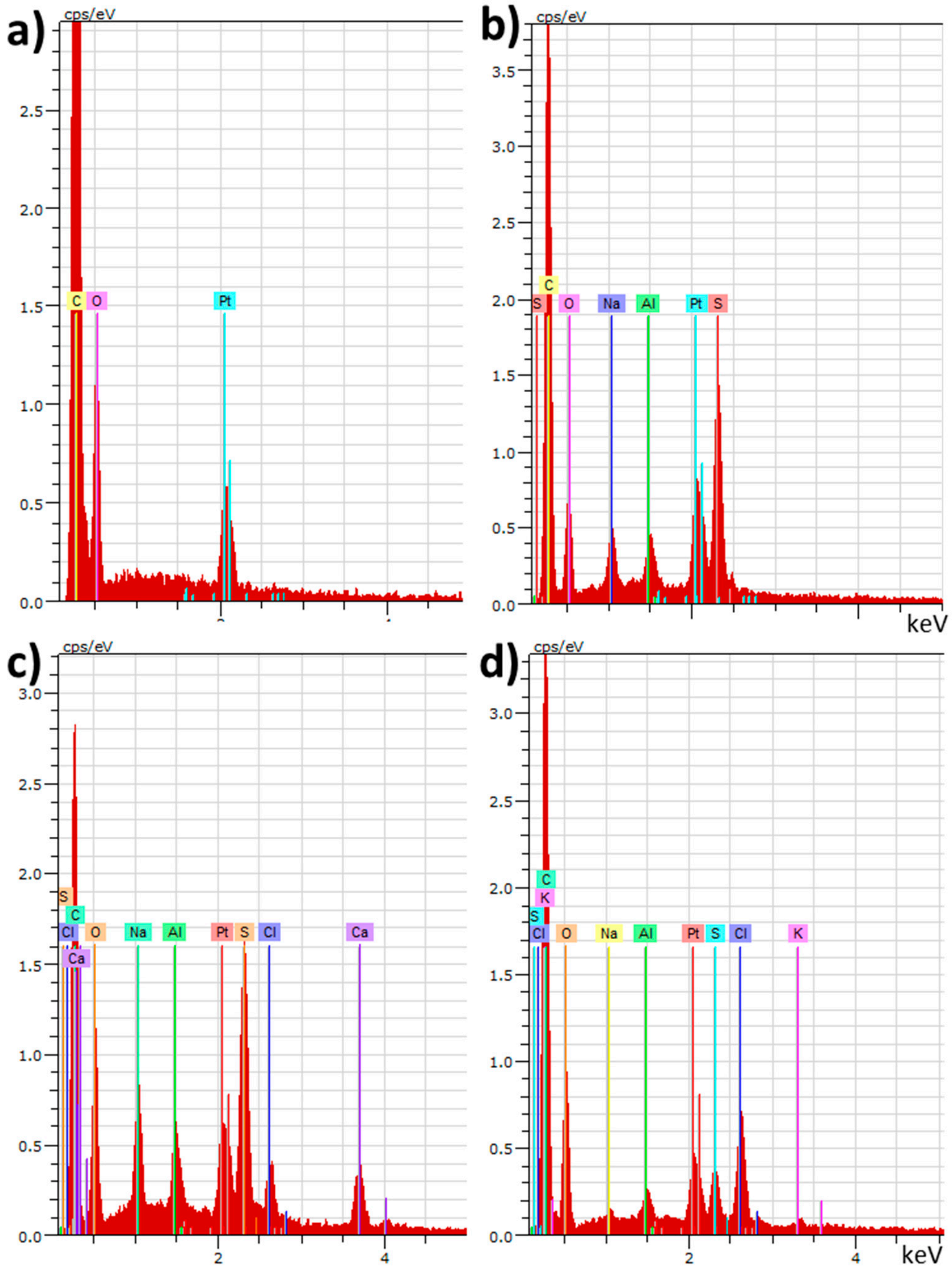

Figure S2. EDS of (a) the uncoated net, (b) the net coated with the 1st layer, P(SSAmC$_{16}$-co-GMA20), (c) the net CN1 coated with P(SSAmC$_{16}$-co-GMA20)/ P(VBCTMAM-co-AA20) and (d) the net CN3 coated with P(SSAmC$_{16}$-co-GMA20)/PAA/PHMG.

In order to investigate the ability of nets of being coated repeatedly with the studied polymeric materials after their use, the system of CN1 was chosen for a re-coating procedure. Thus, the net CN1 which was washed with high pressure water after the first experiment under accelerated lab conditions was re-coated using the same procedure (described in Section 3.3 of the manuscript). The characterization of the re-coated net was carried out by ATR-FTIR spectroscopy, through which the successful modification of the net by this method was

certified. More specifically, Figure S3 shows the spectrum of the net modified with the CN1 system: P(SSAmC16-co-GMA20)/P(VBCTMAM-co-AA20) 55/45 % wt. The graph also shows the spectra of P(SSAmC16-co-GMA20), P(VBCTMAM-co-AA20) copolymers, the washed net and the uncoated net for comparison. As can be seen, the characteristic peaks of the individual copolymers are also observed in the spectrum of the re-coated net. Indicatively, in the first layer the peaks of the methyl groups of SSAmC16 are observed at 2920 and 2840 cm^{-1}, while in the second layer a peak at 1703 cm^{-1} owed to the carbonyl group of acrylic acid is observed. By weighing the net it was found that the loading percentage was 22 % wt., whereas from the photographs of the net before and after the re-coating procedure, no significant changes were observed except of a darkening in the color of the coated net.

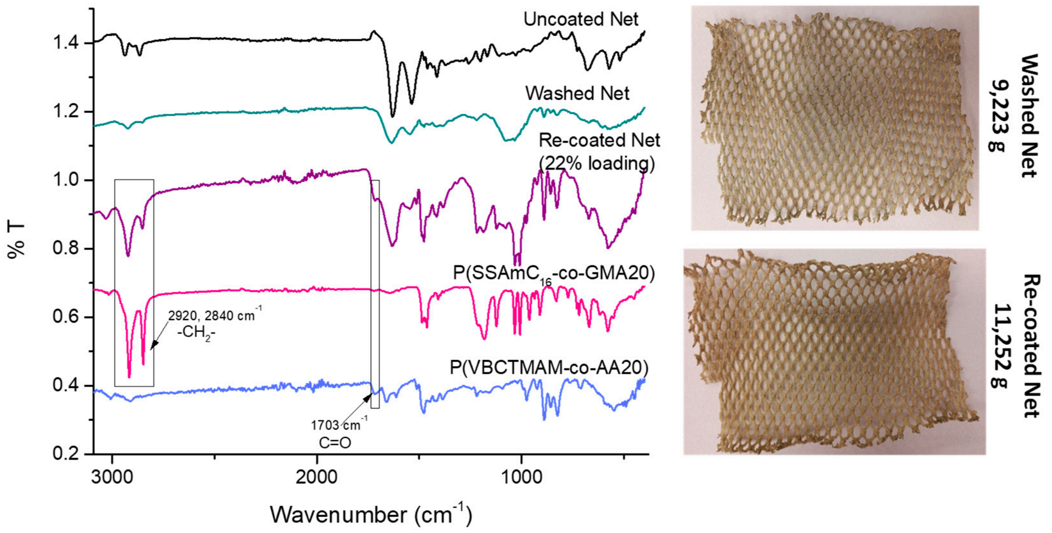

Figure S3. Repeat of the coating procedure on the used net CN1 (P(SSAmC16-co-GMA20)/P(VBCTMAM-co-AA20)) after pressure washing with water, on the 24th day of the accelerated lab experiment. ATR-FTIR spectra (left) and photographs (right) of the washed net as well as the re-coated net for comparison.
